# Editorials

**Published:** 1911-11

**Authors:** 


### EDITORIALS

The Business Office of the *Journal-Record* is 1 1-2 to 5 1-2 South Broad Street.
The Editorial Office is 1014-15 Century Building.
Address all Business Communications to *Journal-Record of Medicine*, 1 1-2 to 5 1-2 South Broad Street.
Make remittances payable *to The Journal-Record of Medicine.*

On matters pertaining to the Editorial and Original Communications, address Edgar G. Ballenger, M. D., Atlanta, Ga.

Reprints of Original Articles will be furnished at cost price. Requests for the same should always be made in THE MANUSCRIPT.

We will present, postpaid, on request, to each contributor of an original article, twenty (20) marked copies of *The Journal-Record of Medicine* containing such article.

### SOUTHERN MEDICAL ASSOCIATION.

The fifth annual meeting of the Southern Medical Association was held at Hattiesburg, Miss., November 14, 15 and 16. In spite of the place being inaccessible to much of the territory, this meeting was conceded by all to have been the most successful one ever held by this Association. Aside from the unusual scientific papers two matters of great importance were decided upon. First, all of the Southern States including the District of Columbia, are now included in the Association. Second, the members agreed to buy the Southern Medical Journal. With this wide territory, needing just such an Association, and with an excellent Journal of its own, very rapid strides should be made by this representative body of Southern physicians. The present constitution does not connect it with any other higher Association. It is strictly an independent organization and now has wonderfully good opportunities in the South for developing and fostering scientific medicine and surgery.

Dr. Isadore Dyer, of New Orleans, was a most satisfactory president, being unusually familiar with parliamentary usages. The writer does not remember to have ever heard a more excellent address than was the one of the retiring president. Dr. J. M. Jackson, of Miami, was elected president for the coming year, and Jacksonville was chosen as the next meeting place. Southern physicians and surgeons should promote their welfare and progress by loyally rallying to this Association.

---

### THE DUST MENACE.

Elsewhere in this issue we publish in full an excellent paper on "The Dust Menace and Municipal Diseases," by Anders. This subject is a most important one which needs more attention. No one living in a city can read Anders' paper and fail to realize the cogency of his reasoning. Here in Atlanta with the excellent drainage on each side of the main streets, we could have the cleanest city in this county if care were taken to wash occasionally and thoroughly the main downtown streets and side streets for a block on each side. When one sees in other cities how well the streets are washed it makes our way of removing only the gross filth seem a farce. The disease-producing germs are chiefly in the dust. If we could have a corps of cleaners to scrub well the streets during hard rains much of the fine dirt and dust which is never removed, except by the drenching rains, could be eliminated. Anders shows the real danger of neglecting these matters.

---

### HEALTH REGULATIONS.

The problem of the care of contagious diseases and our protection from them through household and institutional isolation are pressing upon our Boards of Health in ever varying degree. The advances in the study of medicine are steady and constantly change our attitude toward these diseases, while quite as regularly the hygienic conditions change under the increasing populations. We are learning new features of germ life every day and serum therapy now in use is beyond the ken of the health laws enacted a short time ago. Repeated examinations show that large groups of people, as in cities, are liable to contract certain diseases and at the same time gradually become immune to others. All th[illegible] the health authorities must recognize and comprehend so that proper regulations shall be made and suitable instructions given to the people.

The profession generally is sometimes in error in its attitude towards quarantine and warning methods pursued by the authorities, in that the conditions are not fully explained to the laity. Misunderstandings arise and criticism is applied that could be avoided. In order to harmonize activities for protection with the peace of mind of the householder, health authorities make every possible concession and it remains for the family doctor to do his utmost in establishing cordial working arrangements for protection of the city.

In Atlanta the Health Board presented to the Fulton County Medical Society the suggestion that houses should no longer be placarded for Typhoid Fever because it is not a contagious disease in the ordinary sense and people coming to the house do not need a warning sign. The Society approved this and also the additional suggestion that an Inspector from the Board of Health should visit every house where there is Typhoid Fever and give the family instructions as to the hygienic surroundings. This inspector would not see the patient or interfere in any way with the doctor.

Another suggestion approved by the Society was that hereafter only Typhoid, Diphtheria, Smallpox and Scarlet Fever be reported to the Board of Health. It seems that these diseases were the only ones with which at present it seemed practicable to deal.

The final suggestion was that cases of Diphtheria be dismissed from quarantine twelve days after clinical symptoms have disappeared, whether the specific germ remains in the faeces or not. This plan has been successful in other cities and seems the best balancing between greatly prolonged isolation, on the one hand, and disobedience of the law on the other.

We cordially approve of every effort tending to unite all citizens in a consistent and continuous struggle against contagious diseases and so give these experimental changes our cordial approval in the hope they will lead to more active measures in the near future. We shall never cease to call attention to that most aggressive disease, Tuberculosis, which thus far baffles every civic effort to control it. Public sentiment is not ready to demand drastic measures on the part of the health authorities and we believe it never will be. The people have become calloused to the diseases ravages and have no realization that it can be exterminated. We express the conviction that every Heath Board in the country should *initiate* measures toward controlling this disease that kills far more each year than any other, thus leading and even forcing people to their own protection.

R. R. D.

---

